# Piloting a minimum data set (MDS) in english care homes: a qualitative study of professional perspectives on implementation and data use

**DOI:** 10.1186/s12877-025-06260-6

**Published:** 2025-08-08

**Authors:** Rachael E. Carroll, Nick Smith, Sinead ER Palmer, Jennifer Kirsty Burton, Adam Lee Gordon, Ann-Marie Towers, Stacey E. Rand, Freya Tracey, Anne Killett, Lucy Webster, Barbara Hanratty, Karen Spilsbury, Gizdem Akdur, Kaat De Corte, Julienne E. Meyer, Liz Jones, Claire Goodman

**Affiliations:** 1 Unit of Injury, Inflammation and Recovery Sciences, School of Medicine, University of Nottingham, Nottingham, UK; 2 Centre for Health Services Studies, University of Kent, Canterbury, UK; 3 Personal Social Services Research Unit, University of Kent, Canterbury, UK; 4 Academic Geriatric Medicine, School of Cardiovascular and Metabolic Health, College of Medical, Veterinary and Life Sciences, University of Glasgow, G31 2ER Glasgow, UK; 5 NIHR Applied Research Collaboration East Midlands, Leicester, UK; 6 NIHR Applied Research Collaboration Kent Surrey and Sussex, Lewes, Sussex, UK; 7 The Health Foundation, London, UK; 8 School of Health Sciences, University of East Anglia, Norfolk, UK; 9 NIHR Applied Research Collaboration East of England, Norwich, UK; 10 Population Health Sciences Institute, Newcastle University, Newcastle upon Tyne, UK; 11 NIHR Applied Research Collaboration North East and North Cumbria, North East and North Cumbria, UK; 12 School of Healthcare, Faculty of Medicine and Health, University of Leeds, Leeds, UK; 13 NIHR Applied Research Collaboration Yorkshire and Humber, Yorkshire and Humber, UK; 14 Centre for Research in Public health and Community Care, University of Hertfordshire, Hatfield, UK; 15 National Care Forum, Coventry, UK

**Keywords:** Care homes, Minimum data set, Quality of life, Measures, Implementation

## Abstract

**Background:**

Digitalisation within English care homes offers potential to make more effective use of substantial data collected by staff during care planning and recording. A pilot minimum data set was co-designed with stakeholders based on two digital care records with additional structured measures. Our objectives were to explore (1) care home staff opinions and experiences of collecting structured measures of quality of life, cognition and function for residents and (2) how a minimum data set data might be used by staff and other professionals interested in care homes.

**Methods:**

Between June and October 2023 focus groups and interviews involving care home staff and Integrated Care System participants from three regions of England were undertaken. Integrated Care System staff work externally from care homes and support commissioning of services for care homes and reviewing data. We used a semi-structured topic guide. Two waves of care home focus groups were conducted after each wave of minimum data set data capture. A single wave of focus groups/interviews were undertaken with Integrated Care System participants. Reflexive thematic analysis was used to develop themes.

**Results:**

Twenty-four staff from 22 care homes and 16 staff from 15 care homes participated in five wave one and four wave two focus groups respectively. Ten Integrated Care System participants from two of three study regions participated in one focus group (seven participants) and three individual interviews. Three themes were developed: the care home context and the importance of a minimum data set for care, appropriateness and relevance of quality of life measures to resident care, and data quality and purpose.

**Conclusions:**

Care home staff can collect structured measures on resident quality of life, function and cognition using digital care records to contribute to a minimum data set. The data generated can inform and enhance resident care. However, implementation is an evolving process requiring support, trust-building and confidence among those collecting and interpreting data and incorporation as part of routine care.

**Supplementary Information:**

The online version contains supplementary material available at 10.1186/s12877-025-06260-6.

## Background

Care home staff in England collect and collate substantial data about people living in the care home to support direct care delivery and to fulfil regulatory and contractual requirements [1]. Care homes in England provide 24-hour care and support for adults with complex needs, encompassing services with and without on-site registered nurses. They form a critical part of the social care system, which includes statutory and privately funded care across communities.

Care homes have statutory responsibilities to report data to their regulator, the Care Quality Commission, including on deaths and adverse events. There are other national reporting duties, such as to the Health and Safety Executive, when circumstances require it. Care homes also provide regular reporting data to local commissioners (local authorities and National Health Service (NHS)) to meet contractual responsibilities. Skills for Care collect the Adult Social Care Workforce Data Set using data submitted by care homes [2]. The Capacity Tracker, introduced during the pandemic, has been developed as an ongoing collection from care homes with mandatory reporting requirements and enforceable fines if data are not provided. The data in the Capacity Tracker are for wider sharing within health and social care [3]. Separately from the data collected within the care home, data about care home residents are collected by primary, secondary and community health services, social care and social work. There is significant variation in how, whether, when, and with whom these data are collected and shared, making them difficult to operationalise at scale [4]. This contrasts with other countries which use minimum data sets (MDSs), such as the US Medicare Minimum Data Set (MDS 3.0) [5] and InterRAI [6], to standardise data collection and collation.

Having standardised data collection and collation using MDS approaches has been considered advantageous to inform care planning, enable benchmarking, support resource allocation and enable research [7, 8]. However, our realist review identified the need for resources and support for care home staff to ensure MDS data are valid and valued by them to support resident care, rather than being viewed as administrative burden [9]. Their pivotal role in data collection and use means staff perspectives and experiences are critical to study.

The COVID-19 pandemic exposed the lack of joined-up data about care home residents, providing impetus to address data gaps [10–12]. Until recently, most care homes used paper records but there has been a shift towards digital approaches and the development of Digital Care Records (DCRs) software [13]. This digitalisation has been accelerated in England by central government targets and resource to enable care home DCRs to feed into national digital social care records [14]. Data collected within DCRs on routine care, health status and sociodemographic information could enable better understanding of residents’ needs and hold the key to understanding population health and wellbeing in care homes [15]. Reusing these routine data for research could also reduce burden on residents and staff and offer an opportunity to use data for a shared purpose with greater understanding of the context of data collection [16]. 

As part of the DACHA study (Developing research resources and minimum data set for care homes adoption and use), we set out to develop and test an MDS for English care homes [17]. This involved a programme of work to identify variables for inclusion through: reviewing the international literature; [18] describing measures used in United Kingdom care home trials; [19] surveying current care home data collection; [1] a realist review on MDS implementation; [9] consulting key stakeholders; [20] and engaging care home residents [21]. We aimed to link routinely collected administrative health data collected by NHS England with data from statutory social care providers to care home DCRs with a small number of additional structured measures related to resident needs and quality of life added to DCR software. Linking these data together aimed to minimise care home staff data collection burden to complete the MDS [22]. The framing of the DACHA study, it’s objectives and the focus of data collection drew on nine core principles for a UK MDS (Supplementary Materials) [16]. 

This study was undertaken during a period of significant changes within the care home sector, responding to challenges of the COVID-19 pandemic including high staff turnover and vacancies across care services, resulting in unmet need [2]. However, this has also provided the impetus to recognise the need to invest and grow research capacity from within social care, through more participatory approaches [23, 24]. 

We have described elsewhere the work to access and link data and a description of the feasibility and completeness of the pilot MDS [22]. Here, our objectives were to explore [1] care home staff opinions and experiences of collecting structured measures of residents’ quality of life, cognition and function to contribute to an MDS and [2] how care home staff and commissioners of services for care homes felt MDS data could be used.

## Methods

Reporting of this research has been informed by the Consolidated Criteria for Reporting Qualitative Research checklist (COREQ) [25]. It is part of the wider mixed-methods DACHA study [17]. 

### Study design

A descriptive qualitative study was undertaken, designed drawing on the principles of implementation theory, nested within a wider mixed-methods study. Focus groups were the primary method of data collection, conducted online with care home staff (Waves One and Two in June and October 2023 respectively) and with Integrated Care System (ICS) participants (Wave Two only) to explore MDS data collection and use. Individual interviews were offered to facilitate ICS staff to participate.

### Setting

Older adult care homes using software from one of two DCR providers, in three geographically and demographically diverse regions of England, each representing an Integrated Care System (ICS), were invited to participate. England has 42 ICSs, health and care partnerships including NHS, local authorities, voluntary sector, and social care providers, responsible for planning, commissioning, and delivering coordinated services [26]. The three ICS sites were selected as geographically disparate, serving populations living with different types and levels of social deprivation, ethnic mix, and social exclusion.

### DACHA study Pilot MDS procedures

Most variables in the pilot MDS used data routinely collected by care home staff and were directly extracted from the DCR (e.g. resident demographics, legal status, height, weight, nutritional needs, skin integrity). Externally held data about recruited residents concerning prescribed medications, healthcare use, diagnoses, care home characteristics and workforce data were obtained.

Additional measures not previously available in DCRs and added to the pilot MDS assessed cognition, functioning and quality of life (QoL) (Table 1). Their addition addressed gaps identified following mapping of existing data items in DCRs against an aspirational MDS to address prevailing concerns of care home stakeholders [17, 27]. Researchers introduced care home staff to the additional measures, provided support in locating them within the software and how, practically, to complete them. No formal training was provided about new measures, as the research was interested in exploring how staff would approach the collection and use of the new measures by themselves.

**Table 1:** Summary of additional measures added to digital care records for care home staff completion

Measurement domain	Tool including reference
Capability wellbeing	Investigating Choice Experiments for the Preferences of Older People (ICEPOP) CAPability instrument (ICECAP-O) [28, 29]
Cognitive impairment	MDS Cognitive Performance Scale [30]
Delirium	Informant Assessment of Geriatric Delirium Scale (I-AGeD) [31]
Dementia-specific Quality of Life	QUALIDEM [32]
Functional independence	Barthel Index [33]
Healthcare Related Quality of Life	EuroQol 5 domain 5 level proxy version (EQ-5D-5 L Proxy 2) [34]
Social care Related Quality of Life	Adult Social Care Outcomes Tool Proxy (ASCOT-Proxy) [35, 36]
	Adult Social Care Outcomes Tool (ASCOT): Anxiety and Low Mood subscale [37]
	Adult Social Care Outcomes Tool (ASCOT): Pain item [37]
	Adult Social Care Survey in England (Question 2a) [38]

Care home staff were asked to complete these additional measures twice during the study; six months apart. They had a four-week period to complete measures at each time point. They used their usual digital devices to collect DCR data (including hand-held devices and Personal Computers). Two time points of data collection were planned to determine how staff engaged with the measures over time and to provide an example of how this data may be collected if these measures were included in a national MDS.

DCR data, including additional measures, were extracted by software providers at these time points and shared with the research team in de-identified form for further analysis. Reporting of Pilot MDS item completion is reported elsewhere [22]. 

### Participant selection

A purposive sampling approach was used to ensure care home staff participants had used the pilot MDS (i.e. the DCR and additional measures). Focus groups were undertaken in English. There were no other exclusion criteria.

Participants were approached by email sent from a researcher, with one reminder email. Integrated Care System (ICS) staff work externally from care homes and support commissioning of services for care homes and reviewing data. ICS participants were recruited based on their work with care homes and social care data in each study region and invited *via* professional connections made during study set up [22]. 

Some care home staff participants were known to the interviewers in a professional context through involvement in the study. ICS participants were not known to interviewers.

### Data collection

Data collection was informed by the Consolidated Framework for Implementation Research (CFIR) [39], focussing on experience of implementing the MDS. Topic guides for each wave were developed, and content adapted after piloting and discussion between researchers (Supplementary materials).

Wave one focus groups discussed participants’ views of the MDS, participation in the study and their experiences completing the additional measures (*focusing on objective one care home staff opinions and experiences of collecting structured measures of residents’ quality of life*,* cognition and function to contribute to an MDS*). For wave two, senior care home staff (managers or their representatives) and ICS participants were separately presented with an example MDS output to facilitate discussion around how this could support care delivery (*focusing on objective two how care home staff and commissioners of services for care homes felt MDS data could be used*).

All focus groups were audio recorded and transcribed. The ten focus groups (nine involving care home staff, one involving ICS participants) lasted 43–90 min (average duration 70 min). The three individual interviews lasted 40–90 min (average duration 60 min). Only researchers and participants were present during focus groups.

While the focus groups conducted in this study met all of Krueger’s criteria for a focus group [40], interaction between participants was, at times, closer to what might be more accurately characterised as a group interview [41]. This was particularly the case in the care home representatives’ groups. While there was some interaction and discussion between care home representatives, most of the interaction in the groups occurred between the care home participants and the focus group facilitator. In contrast, the presentation of an example MDS output to ICS participants enabled active engagement, interpretation and reflection and peer-peer interaction within the focus group that went beyond ICS participants’ experience, predominantly, of working with care homes to resolve concerns or difficulties in care provision. In the text we refer to all the groups as focus groups.

#### Data analysis

Reflexive thematic analysis was undertaken using the six stages described by Braun and Clarke [42]. This approach was chosen for its flexibility, particularly in moving between inductive and deductive coding and theme development. The analysis adopted an experiential orientation to understanding the data, with a focus on the experiences and perspectives of care home staff and ICS participants.

##### Familiarisation

Verbatim transcripts were checked by the interviewer/focus group facilitator and uploaded to NVivo (version R1). Thereafter, researchers familiarised themselves through multiple re-reading. Each transcript was reviewed by at least two of three researchers conducting the analysis (REC, SERP, and NS).

##### Coding

Discussion following this initial familiarisation among the project team, led to the adoption of a deductive coding framework drawn from implementation science; the CFIR [39]. The five CFIR sub-domains (innovation, outer setting, inner setting, individual domains, implementation process) were used to enable preliminary organisation of the data. This organisation enabled a focus on key aspects necessary for successful implementation and recognition of where sub domains were absent/not represented. Some sub-domains were frequently used, for example, sub-domain ‘implementation process’, under the construct ‘reflecting and evaluating’ was commonly coded to. This construct was further divided in ‘implementation’ and ‘innovation’ to understand nuances. Initial coding, utilised a semantic coding approach [43]. The coders (RC, SERP, NS) met regularly, including discussing differences in coding and interpretation of any doubled coded transcripts. These sessions focused not on trying to find consensus and a single meaning, but rather to explore the range of different meanings to achieve a richer understanding of the data together.

##### Generating initial themes

The first attempt at moving from codes to themes continued the deductive approach and adherence to the CFIR framework, at this stage using CFIR sub-domains to inform the themes. However, these were felt to be too restrictive to capture the rich experiences shared, thus a decision was made to take a more inductive approach to developing themes from the coding. This identified and mapped recurring ideas, and patterns of experiences that could be tested in other accounts and drawn out as themes.

##### Developing and reviewing themes

Development and revision of the inductive themes involved a wider set of researchers (REC, SERP, NS, ALG, JKB, AMT & CG) who increasingly adopted a more interpretive and latent approach to the data. The team discussed, reflected on, and revised the themes multiple times.

##### Refining, defining and naming themes

Proposed themes were shared within the team and naming was revised to ensure shared meaning, clarity of the relationship between and boundaries of themes. To highlight the non-linear nature of the analytical process, some of these revisions were followed by not only initial attempts at, defining and naming the themes, but also attempts to produce a draft reporting of the findings.

##### Writing-up

This was led by two researchers (REC and NS) with discussion and revisions from others (JKB, ALG, CG, and AMT). The final iterations were reviewed by the entire study team and revised in response to comments received.

#### Research team and reflexivity

Data collection was undertaken by two female researchers experienced in facilitating interviews and focus groups (REC and SERP). REC is a postdoctoral clinical academic with a background in mental health nursing. SERP is a social care researcher with a background in psychology.

Data analysis was led by NS, REC and SERP. NS is a male social care researcher from a social policy and sociology background. Analysis was supported by ALG, AMT, JKB and CG, academics working with care homes.

## Results

### Participants

From 45 care homes recruited to the DACHA study [22], twenty-seven care homes (60%) were represented in focus groups, of which ten care homes were represented in both waves. Twenty-four staff representing 22 care homes participated in five wave one focus groups (Table 2). Groups included between two and nine participants, grouped according to care home type (e.g. single/multiple care home providers) or staff role. Sixteen senior staff representing 15 care homes participated in four wave two focus groups (Table 2). Groups included between two and six participants. A further 25 interested individuals (13 and 12 from wave one and wave two respectively) cancelled due to unforeseen commitments within their care home.

**Table 2:** Summary of care home staff participant characteristics per wave

	**Wave 1**	**Wave 2**
	*Five focus groups* *24 participants*	*Four focus groups* *16 participants*
	**Number of participants** (%)	**Number of participants** (%)
Sex		
Female	22 (91.7)	16 (100.0)
Male	2 (8.3)	0
Age group		
21-30 years	2 (8.3)	2 (12.5)
31-40 years	10 (41.7)	8 (50.0)
41-50 years	6 (25.0)	3 (18.8)
51-60 years	3 (12.5)	3 (18.8)
60 years and over	3 (12.5)	0
Ethnicity		
White ethnic group	15 (62.5)	12 (75.0)
Asian/Asian British	7 (29.2)	1 (6.3)
Black/Black British	0	1 (6.3)
Other ethnic group	2 (8.3)	2 (12.5)
Years in workplace		
Mean [Standard Deviation]	3.7 [3.5]	3.7 [5.8]
Job title		
Care home manager/director	13 (54.2)	9 (56.3)
Deputy manager	4 (16.7)	4 (25.0)
Registered nurse	3 (12.5)	0
Activities co-ordinator	1 (4.2)	1 (6.3)
Administrator	2 (8.3)	0
Senior care assistant	1 (4.2)	0
Team lead	0	1 (6.3)
Clinical service manager	0	1 (6.3)

Ten ICS participants from two of the three study regions participated in three individual interviews and one focus group (seven participants).

While many managers volunteered their care homes’ involvement, in larger chains, participation could be determined by senior staff working remotely from the home, which may have influenced within home engagement in data collection. Some managers, new to post, inherited a decision to participate from a predecessor. In these situations, researchers provided additional support to explain and promote the study and stress participation was an individual’s choice.

The additional measures added to the DCR were not routinely collected and represented new work for care home staff to input. Experience of completing the additional measures ranged from the home manager completing them for all residents, to named staff members being allocated the task, taking account of concerns including workload, consistency of approach and accuracy.

### Overview of themes

**Fig. 1:**
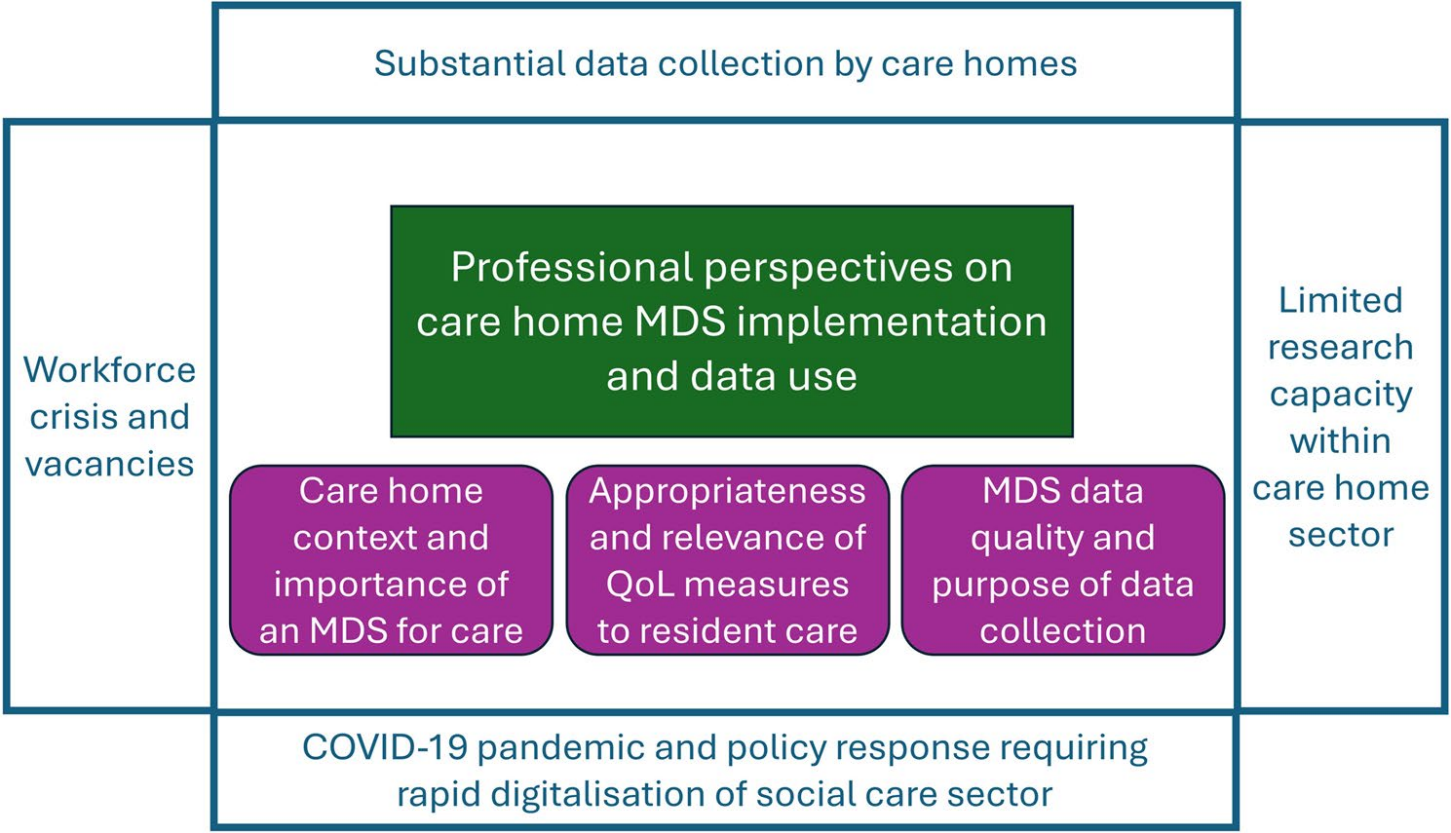
Summary overview of study themes set in wider context of practice Footnotes: MDS– minimum data set; QoL– quality of life

Three main themes were developed from the data concerning professional perspectives on implementation and use of data from the pilot MDS: care home context and the importance of an MDS for care; appropriateness and relevance of QoL measures to resident care; and MDS data quality and purpose of data collection (see Fig. 1).

### Care home context and the importance of an MDS for care

Care home staff operated against a backdrop of workforce pressures and rapid post pandemic change towards digitalisation and reporting. Care homes from one chain had introduced new DCR Software in the year leading up to the research project. Comments to the research team suggested that for some staff completing the MDS was affected by how familiar they were using DCR. Staff turnover during the study period influenced uptake, for example, when managers and/or staff members co-ordinating study involvement in some of the care homes changed. This affected how the MDS was prioritised. The most marked example was that a large corporate chain of care homes withdrew from the study because of a change in ownership and changes in how their DCR software was implemented. Elsewhere, completion was influenced by the equipment, digital literacy, and the culture of how residents’ needs were documented for example, if DCRs were the basis for staff handovers and discussion of residents’ needs. This staff member from the outset could anticipate how the MDS would be incorporated into the routine of the care home:

…*I think if it was to be rolled out*,* it would just get included in the monthly review of the care plans. It wouldn’t be our nurses saying*,* well*,* I’m not doing that bit*,* it would actually form part of the monthly or the review of the care plan (Deputy Manager 4*, Wave One *Focus Group)*.

Staff accounts reflected an overlay between the experience of implementing the care home part of the MDS, and the experience of being involved in a research study. There was willingness to support the research, but without mandate or additional resources for staff time, even with researcher support, managers had to prioritise front line care. These, to an extent, reflect the wider experience of conducting research in care homes, but also reflect the challenges that would accompany MDS implementation in real life. For those who did persevere and became familiar with the format the inputting required was considered to be simple. It was an artefact of the study timeline that to ensure comparable and meaningful data were extracted for all participating residents, care homes needed to collect, collate, and enter additional measures within a month. One participant noted that if collecting these measures became routine, completion could be spread out and more manageable.

*It was just the fact that we were getting so much information in a short space of time. If it was somebody moving into the home*,* you’d be doing that set of questions with them and then reviewing it every month on more of a one-to-one basis. Rather than it being like right*,* I need all 30 people to answer these questions now (Manager 18*, Wave Two *Focus Group)*.

If data capture became routine, and data were entered contemporaneously, then care home staff saw the possibility that data could inform resident care planning forming a positive-feedback loop. Staff would be motivated by the available data and the ways in which it had helped them to deliver and improve care. This, in turn, would lead staff to emphasise the importance of data entry as part of their wider routine. However, this was threatened by the specification and availability of digital and handheld devices and the accessibility of residents’ data. Some of the additional measures were too detailed for a small screen and required a desktop computer. Desktops, meanwhile, were limited in number, sometimes in places away from direct care and could be used by multiple staff for different competing activities. The ability to access information and data when needed during care delivery was suggested as a likely reinforcer of completion if an MDS became routine – but only if these issues of data accessibility and device availability were addressed:

*Hopefully*,* by having easier access to information and quicker access*,* rather than having to kind of ring round the houses to find out information that hopefully would*,* you know*,* you could just log on to a system and see there. (Manager 5*, Wave One *Focus Group)*

There were wider concerns about how the sector could address worries about knowledge, skills, and confidence of care home staff, ensuring staff were equipped to administer the care home element of the MDS. Training was seen as one way to address this:

*A bit more teaching of how to use the information*,* how to extract the information….And then hopefully getting them to understand it’s not a foreign concept and yes*,* it can be adopted*,* or adapted in care homes I should say. (Manager 18*, Wave Two *Focus Group)*

The number of residents lacking mental capacity placed pressure on how resident data were collected and who was accountable. Managers and staff felt responsible for making decisions in residents’ best interests and ensuring ‘the right people’ were involved. This seemed to be a particular concern regarding QoL measures with staff acting as a resident proxy. There were two issues: whether these fitted with their understanding of how to represent the resident; and staff confidence completing new measures. In some cases, this related to the quantitative nature of the data. Thinking ahead, this manager could see staff would need support as part of phased implementation:

*You know*,* some of the decisions we’re inputting*,* it has to be clear we’re inputting as a best interest rather than the individual….So it is more of our views*,* the relative view*,* and it’s really*,* unless they have a full mental capacity that it’s quite difficult to capture that. (Manager 8*, Wave One *Focus Group)*

*So*,* I think everyone at the start will be nervous about it …. anytime I throw numbers*,* even if they have to do observations*,* they’re like oh*,* oh I’m not sure. So*,* you would have to really take small steps introducing it. (Manager 18*, Wave Two *Focus Group)*

### Appropriateness and relevance of QoL measures to resident care

Within the focus groups participants were free to discuss any of the additional measures which were added to create the pilot MDS. However, discussions were dominated by the completion and use of the quality of life measures, which were entirely new to care home staff. Measures of cognition and function were part of existing practice, but in less-structured formats.

Care home staff highlighted that QoL measure content directly affected their completion and use, especially the perceived appropriateness of some QoL items, whether they felt it possible to quantify QoL, and whether and how staff should or could address the domains included.

Though the pilot did not recruit residents considered to be at the end-of-life, concerns about the appropriateness of asking older or unwell residents about their QoL were shared. This raised an important issue around which measures are collected and when, given the short time that many people spend living in care homes. Whether carers felt they could or should respond to QoL domains influenced staff engagement with the MDS. One participant was uncomfortable about the ICECAP-O item on love and friendship and its measurability. Appropriateness was challenged, whilst they accepted, they were responsible for enjoyment (another ICECAP-O item), they questioned if ensuring love should be care homes’ responsibility:

*The group of people we look after are of a very old age with a lot of end-of-life care here and sometimes it can feel a bit inappropriate to ask these questions as well*,* like how do you feel? Well*,* how do you want me to feel*,* I’m dying [laugh]. (Manager 6*, Wave One *Focus Group)*

*I found it’s quite hard to measure love. That might just be how someone feels and that*,* affected by whether they’ve got family*,* whether they’ve got a good relationship with their family and things like that. Enjoyment sort of feels like it’s more our responsibility*,* if that makes sense.* (*Manager 17*, Wave Two *Focus Group)*

ICS participants highlighted wider influences when interpreting data on QoL of residents which need to be considered. They recognised the value of reframing care to focus on the person, their individual needs and perspectives, bringing in concepts of person- and relationship-centred care. For example, enabling care homes to evidence the rationale for care plans that reinforced positive risk taking and acknowledging what they can and cannot influence such as how someone feels about being in a care home.

Care home staff reflected how some questions, such as the ICECAP-O item ‘thinking about the future’, could trigger different ways of discussing care by prompting conversations about the future not solely about preferences and priorities for end of life. It encouraged staff to consider what could be done to improve QoL for residents in the present:

*that person’s dignity*,* their control over their life*,* safety*,* it [the QoL measure] all presumes that that comes from the care home. But actually*,* we [ICS commissioners] know that there are multiple factors that impact on a person’s safety and risk taking…….we’ve got to see people as that holistic being*,* with multiple factors impacting on them. (ICS participant 3*,* Focus Group)*

*If I’m honest in terms of a care home*,* future planning and future preparing*,* all we do is look at what people’s wishes are at the end of their life……There’s not as much that we do in terms of well actually*,* what do you want to do….So yeah*,* shines a bit of a light on how we can look at that differently*. (*Manager 17*, Wave Two *Focus Group*)

Believing data could impact on residents QoL was seen as key facilitator towards completion – triangulating with the point made earlier about positive feedback loops inspiring data completion. This was also the case where data informed care home teams ideas about what they were good at, and where further training and staff development were needed.

…*maybe if it’s something you had on a monthly basis*,* you’d be able to then look ahead and say right*,* well*,* we’ve got this training planned*,* is there something that we need to add in because this has all of a sudden shot up? (Manager 7*, Wave Two *Focus Group)*

Several care home participants discussed how QoL measures fostered a wider view of care beyond medical or physical needs, or environment. Care home participants suggested that QoL measures, ASCOT-Proxy in particular, not only provided a more structured in-depth account of residents’ lives but could also facilitate person-centred approaches to care that enhanced their care work involving staff and providing opportunities for family and friends to contribute:

….*we can sit with them and discuss things with them that we wouldn’t normally discuss with them. The care plans*,* on a whole are becoming so person centred it warms my heart. Our residents are getting much*,* much better looked after*,* and this is all because of the DACHA study…. It’s changed the way I look at things*,* and I’m sure if CQC comes out we’ll be able to say to them*,* look*,* we’ve been involved in this study and we’ve noticed that this has improved our level of care*,* and the input from our residents and their families*.

*It would definitely contribute to the evidence that we would be providing to CQC to say what we do is outstanding. It’s definitely what we would use. Because a lot in (Software name) as well*,* all the risk assessments and stuff that we already do on there are very*,* very clinical. It’s all based on how someone*,* how your body works*,* that’s what it is. And so….adding all of these additional things in*,* we’re looking at people holistically*,* it’s so person centred*,* it’s beautiful. (Manager 16 and Manager 17*, Wave Two *Focus Group)*

There was evidence that participating care homes used the QoL data to inform changes in practice. Some participants were hopeful that continued use could positively impact residents’ lives. They recognised the process did not necessarily lead to “answers” but triggered important conversations. Part of this was that QoL data could present details about how residents regarded their lives, or day-to-day care, that care home staff found distressing, but that they also found it important to hear to deliver care that met residents’ needs.

*This is quite heart breaking for me in the setting that I work in*,* quite devastating. How do you feel about your future*,* when someone can’t remember what day of the week it….it’s great that we are capturing what’s important to people*,* but this was quite difficult. (Manager 4*, Wave One *Focus Group)*

Balanced against these views was the sense, for some participants that quantitative summaries of QoL were reductive and driven more by measurement than care concerns. For participants who espoused these views, they couldn’t cite examples from their own care homes where QoL data had facilitated reflection or improvement. It is not clear the extent to which negative baseline views about QoL measures cultivated disengagement, or whether these staff had tried to work with the measures and found them of limited use in practice.

*MDS data quality and purpose of data collection*.

Care home and ICS participants recognised the importance of data quality, as it would influence uptake and data use. Care staff are expected to deliver care and it is a role that carries much responsibility. Managers expressed concerns over the expectations being placed on a low-paid workforce:

*you want all that accurate data straight away. But I mean this with no disrespect to absolutely anybody*,* we employ an unskilled labour force that are being paid minimum wage*,* and yet we want the highest of standards in the most regulated industry that I know of. (Manager 11*, Wave One *Focus Group)*

There was an extended discussion between ICS participants that they needed to be more trusting, recognising that their opinions were often prejudiced by having to focus on the (minority) of care homes who were not performing well. This related to two areas of data collection, reporting on residents’ unmet need and the use of proxy assessments.

For some ICS participants, what emerged was a belief that care homes and their staff were likely to be biased in their reporting. They wondered if care home staff might feel exposed by highlighting unmet needs and be tempted to frame data entry in a way that portrayed care in the home in a positive light. This was based largely upon supposition and no respondents gave examples where care homes had done this in the past.

. *it’s going to reflect on the overall care of the care home. So*,* part of me would be questioning the validity of the data from the care staff who’ve completed it. You’re not going to want to say that they’ve got safety unmet needs in a care home necessarily.* (*ICS participant 1*,* Interview*)

The ICS focus groups also expressed concerns about using proxies to understand QoL, with a preference for measures based on resident self-report QoL, exposing a lack of understanding about care home residents’ capacity to complete such measures. Balanced against this was a recognition that data about care home residents’ lives, experiences and QoL are scarce. Proxy data are better than the current inability to meaningfully describe these:

*It’s not like we’re not trusting them or not that we think that they’re fudging the data or the results. I just don’t understand how you can make that analysis of somebody else’s quality of life….Even if you ask my partner*,* my family*,* what they would say*,* and the answers they would give would be different to what I would give. (ICS participant 8*,* Focus Group)*

*QoL metrics….are something that are lacking within datasets*,* not just in care homes*,* I think generally across data that we collect. So*,* I think this is very welcome. (ICS participant 9*,* Interview)*

Care home staff shared this sense that proxies somehow fell short of the aspiration of individualised person-centered care. There were examples of staff despite being asked to complete QoL measures in a resident-proxy format, describing how they would engage in additional conversations with residents to try and validate their interpretation of the measures.

Despite ICS participants’ concerns around data accuracy, they were able to propose ways data could add value to care. An example around aggregating resident level data to describe the health of the local care home population, and how they used hospitals services over time, illustrated how an MDS could support wider ICS work on population health, resource use and trends. Both care home and ICS participants understood that an MDS has different purposes for different people. For care home representatives, a secondary use of the data could be to evidence their interactions with other parts of the health and social care system. For example, data from the MDS could demonstrate a care home requires more support from visiting health services. These two quotes from an ICS and care home staff participant respectively, demonstrate how aggregate data could inform service review:

*I think most ICSs are doing*,* is looking at population health management. So*,* this isn’t about necessarily tackling individual residents and the issues that they face*,* but it’s looking at care homes. And actually*,* are there clusters of care homes*,* and a lot of them are provided by the same provider for instance*,* or in the same patch*,* or they have the same GP linked to them*,* for instance. (ICS participant 9*,* Interview)*

…*that could be a possibility*,* to start the conversation with your local authority*,* CCG to actually identify that there is a lack here*,* the GP is not supporting….We can have a conversation and evidence to say that this is the problem comparatively. You know*,* our home is not receiving the sufficient support*,* so is there anything that you can actually bring us*,* give us*,* something along those lines*,* yeah. (Manager 9*, Wave Two *Focus Group)*

Both care home and ICS participants said that care homes would want to use data to show regulators how well they supported residents, supporting the case their home provided outstanding care:

*It’s really hard to say what you do and how you do it and be able to prove that.… I’m thinking for the homes and the workforce in that home*,* to be able to go and say actually we do a really good job. Our score reflects that we are good at what we do. (ICS participant 5*,* Focus Group)*

Using data outside of care homes to create national and local averages would, according to ICS participants, enable benchmarking against other providers. It was hoped this could stimulate *“a bit of healthy competition” (ICS participant 9*,* Interview)* and improve performance across the sector. Other ICS participants suggested that QoL could be seen as a marker of care quality and used by the public to value care homes.

*If [MDS data] went to the public domain……to say that they’ve got a two [for] QoL or whatever. Then actually*,* I’d look at CQC rating and it’d be good to have that as well….So*,* I think we do compare at the minute just on that ratings and I think it would be good to understand*,* to have a QoL [score] would be great for all of us. (ICS participant 3*,* Focus Group)*

There was discussion of using MDS data to sanction or motivate care homes. The data were attractive to ICSs who were considering how to support quality in their regions. A focus on quality improvement, however, was defined more negatively as *“scrutiny”* by another ICS participant (*ICS participant 4*,* Focus Group*) demonstrating how underlying beliefs and assumptions about the role of ICS when working with care homes could affect how MDS findings are perceived.

There were contrasting views about if the data should be widely available. Using data to make judgements without understanding the context of care or the variation of provision was a recurring concern for some care home participants:

*I think if it’s freely available*,* I think that’s not really good. I think if you’re able to take different aspects of it to use to promote or develop your care and things*,* that’s fine. But not for all and sundry to have full access to everything*,* because it’s judgments again*,* isn’t it? It’s making those judgments on the different areas*,* different homes. (Manager 15*, Wave Two *Focus Group)*

## Discussion

### Findings in context

Within a mixed-methods study evaluating a pilot MDS in English care homes we explored care home staff professional perspectives on collecting new structured measures within existing DCR software. Additionally, we explored how senior care home staff and commissioners of services for care homes felt MDS data could be used. The insights shared within our themes, particularly around the measurement of resident quality of life, should inform plans for national data collection involving care homes.

Data collection in care homes is often represented as administrative work, externally imposed, distracting from core caring responsibilities [44, 45]. Participants’ accounts in this study provided evidence of circumstances supporting culture shift to one where resident data informs day-to-day care. A systematic approach to collecting data on residents’ characteristics and QoL could increase staff awareness and directly inform care planning and delivery. For those care homes who persevered with data capture they identified benefits for individual residents and increased job satisfaction in being able to link care work to how it was documented and discussed. The process of data capture was valued when it enabled identifying residents’ preferences and priorities, evidencing the care staff deliver and identifying areas for improvement. We found that meaningful change required senior staff involvement, a level of organisational readiness, shared confidence in validity of the data, and a belief that new systems aligned with existing organisational values. Data use beyond the care home required trust in data capture and interpretation which is aware of the diversity of care home residents, their capacity to articulate their needs and the circumstances of the care homes which support them. While there is a potential value in comparing services and enabling benchmarking, there is a need for data to be adjusted to account for differences in the resident population which may impact findings [46]. 

The implementation process relied on staff interest and encouragement from researchers and evidence of direct benefit to the resident and staff supporting them. Despite this, some care homes stopped collecting data. Sustaining consistent data capture and use requires assessment of staff availability, engagement, and IT resources. Ongoing training needs to reinforce the importance of different data categories, underpinned by opportunities to discuss the evidence within and beyond the care home. These findings are consistent with prior work implementing innovation in care homes [47, 48], where health and social care organisations and their staff have established ways of working together based on trust and parity of esteem [8, 49, 50]. 

The discussions about who completes, uses and shares MDS information, underline the need to pay more attention to systems of working within care homes and invest in activities that foster a sense of shared responsibility within care homes and with external partners. The narrative of digitalisation and integration needs to be explicit about the work required to achieve this.

Our review of MDS implementation internationally found mandates and incentives were key, complemented by bottom-up and top-down approaches for effective implementation [9]. Recent experiences of policy mandates and incentivised care home data capture during the COVID-19 pandemic (the Capacity Tracker) suggest that this alone does not guarantee data completeness or quality [47, 51]. Incremental, reflective approaches, involving senior care home staff as implementation leaders, focussing on continuity and collaboration are also needed [52, 53]. To reduce workload and duplication of effort, all stakeholders must be confident that the MDS is sufficient to address their information needs, with additions collectively negotiated and agreed.

The researchers did not impose a specific approach with care home staff to complete additional measures. Instead, the study made these available to staff, provided support with practical issues, then observed and reflected on how staff collected and used the data. The experiences shared provide evidence of the potential value added by the data to influence resident care, enhance staff interactions and increase job satisfaction. Some staff found it difficult to explore topics where residents reflected negatively on their own QoL or overtly acknowledged their own limited life expectancy, within our data and our public engagement involving residents [21]. These discussions are important given the vulnerabilities and reality for many people living in care homes in their average shorter survival compared to age-matched peers living elsewhere in the community and varied survival time after moving-in [54, 55]. It is critical that measures to evaluate QoL among care home residents are sensitive to the setting, the population and the care which is in place to support the individual. As discussed, this is population with significant and often changing health needs, which will influence their QoL. Additional analysis found that the Adult Social Care Outcomes Toolkit (ASCOT) was associated with care quality (determined by the care regulator) and effectiveness (avoidable hospital admissions) [56]. In a population experiencing ever declining health, maintaining QoL should be the goal – and we need measures that are sensitive enough to do this and do not simply reflect individual’s functioning. Interpreting and using these data requires understanding of the context in which they have been collected. Other measures of personal outcomes have been developed but so far not tested more widely in care home settings [57]. The findings demonstrated how QoL measures could inform staff conversations about residents’ priorities and experiences of care. Staff engagement in QoL measures as part of an MDS could be an important way to surface residents’ views using them to inform and personalise their care, enhancing relationships, known to enhance care quality [58]. Future research is needed to explore whether normalising the use of these measures leads to sustained changes in assessment and practice.

To enable participation and systematic evaluation of QoL among the care home population, there is a need to use proxy measures [27, 59]. Despite evidence of validity this was not universally accepted by data users. Education and support is required around this, appreciating proxy measures are not intended as equivalent to those from individuals, but a valid way to offer insight into the QoL of those who would otherwise be excluded [56, 60]. 

Reflecting on the data overall, we identify learning point to inform MDS implementation in Table 3 and have identified six research questions to inform a national roll-out in Table 4.

**Table 3:** Learning points to support care home MDS implementation

• Planned data collection periods that reflect care home priorities and routines
• Resource to support additional data gathering
• Training to support staff familiarisation, confidence and adoption of new measures
• Adequate IT facilities and fit between the hardware and software to enable the completion of simple and more complex assessments close to the older person
• Ability for data to influence residents’ care planning
• Care home leadership engaged with data capture and supports the embedding of practices that enable review and discussion of MDS
• Care home staff, commissioners and visiting professionals develop ways of working that reference MDS findings
• Resources and policy briefings that promote a shared understanding of QoL outcome measures. Specifically, how they can inform practice, commissioning and evaluation, recognising when differences in residents’ profiles are due to factors outside of the care homes' control.

**Table 4:** Questions arising from findings requiring further exploration before widespread implementation

1. How do staff respond, in terms of their feelings and care actions, following structured evaluation of a residents’ quality of life?
2. What support do staff need after hearing difficult responses around quality of life among the residents they care for?
3. What is the optimal timing and frequency of MDS data collection, focusing on the perspective of influencing direct care?
4. How can software providers best integrate new measures into their products to make quality of life information accessible to frontline care staff and other data users (e.g. accessing current/previous assessments, longitudinal data and trends at a resident and care home level)?
5. What information and training do stakeholders need to facilitate their interpretation and use of MDS data collected about the care homes they support?
6. How will longitudinal data be interpreted and used if quality of life becomes routinely evaluated in English care homes?

## Strengths & limitations

A key strength of the work is drawing on care home staff experience of collecting resident data on QoL, cognition and function to add to the MDS and using the data these generated in their practice. In addition, using pilot MDS data to facilitate discussion with ICS participants and care home staff enabled exploration of potential data use. This facilitated authentic testimony based upon lived experience. Discussions were further focussed onto areas relevant to implementation by using CFIR as an organising principle, with a shift to more inductive analysis to capture the richness of individual experiences. That discussion focused on the QoL measures, rather than those for function or cognition is noteworthy. There was enthusiasm for the QoL measures, which captured something novel, revealing insights or evidence not previously shared. In common with our consultation findings, structured evaluation of QoL was considered a priority with the pilot MDS [20]. Our work was undertaken, consistent with the underpinning principles developed, with a focus on measuring what matters most, co-producing evidence based content with stakeholders and embedding data collection within DCRs [16]. 

We acknowledge the potential selection bias associated with including those who completed the additional measures in our sample and not those who did not. There are questions around care homes’ representativeness in terms of their capacity to take on additional work and we acknowledge that the enthusiasm of staff for the additional measures is likely to have contributed to participation [47]. Our care home participants were predominantly senior staff, as they took the lead on data completion, thus the perspectives of care staff are under-represented within the workforce. We did not capture the perspectives of staff unable to participate in focus groups conducted in English. Focus group size was heterogeneous as not all care home staff were able to attend as planned due to competing pressures within the care home. It was deemed preferable to proceed with groups, rather than cancelling, to facilitate their participation. In one study region, no ICS participants were recruited, so their perspectives are missing. Finally, we recognise care homes were at different stages in terms of familiarity with DCR software and the study then altered this content. This represents a change from usual activity, but then so would de novo implementation of an MDS in real-world settings.

## Conclusions

Our data show that care home staff can complete structured measures assessing residents QoL, function and cognition to contribute to a pilot MDS using existing DCR software. When they do this, they can see the potential use of the data generated by their assessments to inform and influence residents’ care to enhance existing practice. Successful implementation requires a cultural shift such that measures are perceived as appropriate and relevant to direct care with resources to sustain their use, enabling data collection to be part of routine care. This should be recognised by policymakers and practitioners as an evolving process that requires senior support, and investment in activities that build trust and confidence of those collecting and interpreting data, mindful of the context in which they were collected.

## Supplementary Information

Supplementary Material 1.

## Data Availability

The data generated and analysed during the current study are not publicly available due to the ethical approvals secured for the study but are available from the corresponding author on reasonable request in de-identified form.

## Abbreviations

ASCOT: Adult Social Care Outcomes Toolkit
CFIR: Consolidated Framework for Implementation Research
COVID-19: Coronavirus disease caused by SARS-CoV-2 virus
DACHA: Developing research resources And minimum data set for Care Homes Adoption and use
DCR: Digital Care Record
ICS: Integrated Care System
MDS: Minimum Data Set
NHS: National Health Service
QoL: Quality of Life
