# Supplementary material for: Piloting a minimum data set (MDS) in english care homes: a qualitative study of professional perspectives on implementation and data use
Source: BMC Geriatr. 2025 Aug 8;25:604. doi: 10.1186/s12877-025-06260-6 (PMC12333298; doi:10.1186/s12877-025-06260-6)
Supplement: Supplementary file 1 — Supplementary Material 1. [file 12877_2025_6260_MOESM1_ESM.docx]

Supplementary Materials

| **Item** | **Page number** |
| --- | --- |
| Core principles for a UK minimum data set for older adult care home residents | 2 |
| Wave One Focus Group Schedule | 3 |
| Wave Two Focus Group Schedule (Care Home Participants) | 5 |
| Wave Two Focus Group/Interview Schedule (ICS Participants) | 6 |

Core principles for a UK minimum data set for older adult care home residents

| 1. The MDS must primarily focus on measuring what matters most to support those living in care homes through systematic data collection and sharing. 2. The MDS must be evidence-based in design and contents, requiring co-production with key stakeholders. 3. The MDS must reduce data burden and duplication of effort for the care home. This will be achieved through piloting, collaboration, and ongoing engagement with homes. 4. The MDS will be most effective when underpinned by digital care planning and care records systems, within the care home, serving the day-to-day needs of residents, staff, families, and friends. This requires digital infrastructure and investment to deliver at scale. 5. The MDS will include information on the care home service, individual-level data on residents, and information on the model of staffing that supports them, but will not include individual-level data identifying the workforce in each home. 6. The MDS should bring together data from within the care home, coupled with data held externally about residents and care services. 7. Data sharing with external users of the MDS must have an agreed purpose. Data sharing pathways must be defined and formalised in data sharing agreements, using secure environments for access where appropriate. Care home residents’ privacy rights must be protected. 8. Care homes should be supported to access and use the data they collect and share using electronic dashboards. 9. The MDS requires national infrastructure and integration with existing data systems. |
| --- |

Reproduced from Burton *et al.* Developing a minimum data set for older adult care homes in the UK: exploring the concept and defining early core principles. Lancet Healthy Longevity. 2022;3(3):E186-93. DOI: 10.1016/S2666-7568(22)00010-1

Wave One Focus Group Schedule

1. **We want to start by asking how much you know about the aims of the DACHA project?**

Prompts: We are aiming to test the introduction of something called a “minimum dataset” – core information about how residents receive their care, which we can draw together to help inform care decisions. Does anybody have any thoughts about this?

1. **What has your role been in the study?**

Prompt: were you involved in the recruitment of residents, admin of the study?

1. **Can you see value in having a minimum data set? How do you think it will be beneficial? To whom? Do you see any harms or challenges associated with it?**
2. **We spent quite a bit of time in the care homes, working to recruit residents to DACHA. This might mirror what we have to do in real-life if a minimum dataset were to become part of routine practice? Do you have any thoughts about this process?**

Prompts: What did you think about how we approached residents/their families?
Could this have been done better, or differently? What were your thoughts on the digital consent process that we used for relatives?
How could we go about incorporating this type of consent into routine practice in care homes?

1. **Additional measures: Most of our minimum dataset comes from information that you already collect routinely as part of your work in the care home. We had to add a few additional to ensure that we provided a full overview of the residents and the care we received. We are now going to put these measures up on some slides, so that you can see them. For each we’d like to ask you what you think about it?**

A slide for each of:

1. DACHA - Barthel Index of Activities of Daily Living
2. DACHA - Adult Social Care Outcomes Toolkit (ASCOT)
3. DACHA - EQ5D-5L
4. DACHA - ICECAP-O Quality of Life Questionnaire
5. DACHA - Informant Assessment of Geriatric Delirium (I-AGeD)
6. DACHA - MDS (Minimum Data Set) Cognitive Performance Scale
7. DACHA - QUALIDEM - Dementia specific quality of life tool
8. DACHA - Self-Report QoL from ASCS

For each of these elicit conversation – possible prompts:

- What do you think of this measure?
- Is it important to residents?
- Is it important to the team providing care
- Is it easy/difficult to collect?
- Do you see any problems with collecting this.
- Will you use this measure outside of the DACHA study?

1. **Who was it that completed the additional measures? How was this decided?**
2. **What sort of device did you use to complete them? Handheld device, desktop, (paper). Were there any issues with/benefits to this device?**
3. **If we’re collecting this type of data routinely, what might make it easier, or more difficult?**
4. **Is there anything else that you’d like to tell us?**

Wave Two Focus Group Schedule (Care Home Participants)

We are going to start by showing you some data that we have collected as part of the minimum dataset from the DACHA study. We’ve worked to formulate this in a number of different ways that might be useful to care home staff. For each of these we want to have your thoughts on whether, and how, it might be useful to you.

1. **Present a series of slides with different data sharing prototypes, for each one consider the following prompts:**
   - What do you think about what’s on this slide?
   - Would this type of data be useful to you?
   - How might you, or other staff in the home, make use of it?
   - What do you think of the presentation?
   - Do you see any limitations to how the data are presented here?
2. **The data in our minimum dataset might be useful to other people outside of the home, for example healthcare professionals, or regulators. What are your thoughts on this?**

- Prompts: highlight different professional groups
  - GPs
  - Community nurses
  - The local authority
  - NHS managers/commissioners
  - The CQC
- Be prepared to prompt on
  - why particular groups would be seen to have more or less of a right to the data
  - what safeguards or reassurances might need to be in place
  - how this might impact upon the readiness, or otherwise, of care home staff to participate in data collection processes

1. **We want to know what makes care home staff enthusiastic, or nervous, about a minimum dataset, can you tell us your thoughts on this?**
2. **Is there anything else that you’d like to tell us?**

Wave Two Focus Group/Interview Schedule (ICS Participants)

I’m going to share with you some prototype data outputs and ask for your thoughts on these:

1. **Present a series of slides with different data sharing prototypes, for each one consider the following prompts:**
   - What do you think about what’s on this slide?
   - Would this type of data be useful to you?
   - Who else in the NHS might want to make use of these data?
   - Are there any other datasets held within the ICS that might compliment or conflict with what is shown here?
   - Do you have any concerns about this type of data coming from care homes?
2. **There are a large number of variables in the minimum dataset and we’ve only presented some of these here to you today. What else might you, or others in the ICS want to learn from a care home minimum dataset?**
3. **Is there any data coming from a care home minimum dataset that you shouldn’t see? Or that you feel should be restricted in who can view? Who should it be restricted to?**
4. **What are the benefits and risks associated with this type of minimum dataset?**
5. **Is there anything else that you’d like to tell us?**
